# High Prevalence of Malaria in Zambezia, Mozambique: The Protective Effect of IRS versus Increased Risks Due to Pig-Keeping and House Construction

**DOI:** 10.1371/journal.pone.0031409

**Published:** 2012-02-20

**Authors:** Emmanuel A. Temu, Mike Coleman, Ana Paula Abilio, Immo Kleinschmidt

**Affiliations:** 1 London School of Hygiene and Tropical Medicine, London, United Kingdom; 2 Liverpool School of Tropical Medicine, Liverpool, United Kingdom; 3 National Institute of Health, Maputo, Mozambique; 4 Tropical Epidemiology Group, London School of Hygiene and Tropical Medicine, London, United Kingdom; Kenya Medical Research Institute - Wellcome Trust Research Programme, Kenya

## Abstract

**Background:**

African countries are scaling up malaria interventions, especially insecticide treated nets (ITN) and indoor residual spraying (IRS), for which ambitious coverage targets have been set. In spite of these efforts infection prevalence remains high in many parts of the continent. This study investigated risk factors for malaria infection in children using three malaria indicator surveys from Zambezia province, Mozambique. The impact of IRS and ITNs, the effects of keeping farm animals and of the construction material of roofs of houses and other potential risk factors associated with malaria infection in children were assessed.

**Methods:**

Cross-sectional community-based surveys were conducted in October of 2006, 2007 and 2008. A total of 8338 children (ages 1–15 years) from 2748 households were included in the study. All children were screened for malaria by rapid diagnostic tests. Caregiver interviews were used to assess household demographic and wealth characteristics and ITN and IRS coverage. Associations between malaria infection, vector control interventions and potential risk factors were assessed.

**Results:**

Overall, the prevalence of malaria infection was 47.8% (95%CI: 38.7%–57.1%) in children 1–15 years of age, less than a quarter of children (23.1%, 95%CI: 19.1%–27.6%) were sleeping under ITN and almost two thirds were living in IRS treated houses (coverage 65.4%, 95%CI: 51.5%–77.0%). Protective factors that were independently associated with malaria infection were: sleeping in an IRS house without sleeping under ITN (Odds Ratio (OR)  = 0.6; 95%CI: 0.4–0.9); additional protection due to sleeping under ITN in an IRS treated house (OR = 0.5; 95%CI: 0.3–0.7) versus sleeping in an unsprayed house without a ITN; and parental education (primary/secondary: OR = 0.6; 95%CI: 0.5–0.7) versus parents with no education. Increased risk of infection was associated with: current fever (OR = 1.2; 95%CI: 1.0–1.5) versus no fever; pig keeping (OR = 3.2; 95%CI: 2.1–4.9) versus not keeping pigs; living in houses with a grass roof (OR = 1.7; 95%CI: 1.3–2.4) versus other roofing materials and bigger household size (8–15 people: OR = 1.6; 95%CI: 1.3–2.1) versus small households (1–4 persons).

**Conclusion:**

Malaria infection among children under 15 years of age in Zambezia remained high but conventional malaria vector control methods, in particular IRS, provided effective means of protection. Household ownership of farm animals, particularly pigs, and living in houses with a grass roof were independently associated with increased risk of infection, even after allowing for household wealth. To reduce the burden of malaria, national control programs need to ensure high coverage of effective IRS and promote the use of ITNs, particularly in households with elevated risks of infection, such as those keeping farm animals, and those with grass roofs.

## Introduction

Malaria, especially that caused by *Plasmodium falciparum*, remains one of the most important causes of morbidity and early mortality in endemic regions of sub-Saharan Africa [1]. A massive scale-up in malaria control programmes between 2008 and 2010 has resulted in the provision of Insecticide Treated Nets (ITNs) to protect more than 578 million people at risk of malaria in this region [1]. Similarly, Indoor Residual Spraying (IRS) has protected 75 million people in 2009. Due to the scaling up of interventions, the number of deaths caused by malaria is estimated to have decreased from 985,000 in 2000 to 781,000 in 2009 [1]. National strategies to monitor malaria interventions include malaria indicator surveys that measure malaria infection prevalence, coverage and usage of various interventions and changes in knowledge attitude and practices in malaria control [2]–[4].

Many of the risk factors for malaria are related to access to interventions and inversely related to a household's socio-economic status (SES) with increasing vulnerability of the poorest [5]. In addition, malaria risk varies widely between localities or even households due to their specific characteristics (locations, household possessions, house construction, farm animal ownership and distribution of mosquito breeding sites) that may facilitate human/mosquito contact increasing the likelihood of malaria infection. In regions where the malaria mosquito vectors feed on both animals and humans, the presence of farm animals close to the household may also affect the risk of malaria transmission to humans. The nature of this impact is likely to be vector and site specific [6].

The African Summit on Roll Back Malaria, held in Abuja, Nigeria in 2000, specified that 60% of population at risk of malaria should use ITNs by 2005, a target that was raised to 80% by 2010 [7]. But use of ITNs remains below the target with only 18.5% of African children in stable malaria transmission areas protected by a net in 2007, leaving nearly 90 million unprotected [8].

During the past decade, IRS has been implemented successfully in Mozambique, Eritrea, Ethiopia, Equatorial Guinea, Island of Principe and Madagascar [9]–[11]. With funding from the President Malaria Initiative (PMI), the Global Fund and the private sector, African countries are expanding ITN and IRS programmes as part of their national malaria control strategies.

In Mozambique malaria is endemic, affecting the entire population of around 23 million people [12] with an estimated 44,000 to 67,000 malaria specific deaths each year across all age groups [13]. Over 90% of reported cases are due to *P falciparum*. Transmission is perennial with peaks during and after rainy seasons. The most important malaria vectors belong to the *Anopheles gambiae* complex and the *An funestus* group [14].

Mozambique is currently promoting ITNs and IRS for malaria prevention. Long lasting insecticidal nets (LLIN), are distributed free to pregnant women and children under five years old. IRS campaign for malaria control has been conducted in Mozambique using various insecticides. Before 2000, pyrethroids were used for IRS but the detection of resistance [14], [15] led to the replacement by carbamate. However due to the high cost of using carbamate for IRS, DDT was introduced in 2005 and used until 2008.From 2009, pyrethroids were again used as main insecticide for IRS [16].

In this study, the effects on malarial infection of vector control interventions, house construction, ownership of farm animals, household wealth and other potential risk factors were investigated using malaria indicator survey data from Zambezia province, Mozambique.

## Materials and Methods

### Study site

Zambezia province is situated in central Mozambique, has an estimated population of 3,794,509 and covers a total area of 103,127 km^2^, much of it drained by the Zambezi River [17]. There is considerable forest inland and much of the coast consists of mangrove swamps. Zambezia province is characterized by a seasonal pattern of rainfall from October to June and malaria is perennial with transmission peaking during the rainy season.

In Zambezia province, IRS with DDT was re-introduced in 2006 through the Mozambique National Malaria Control Program (MNMCP), supported by the US Presidents Malaria Initiative [16]. In 2009 there was a change in the insecticide from DDT to pyrethroids. Pyrethroid resistance in *An. funestus* was first reported in southern Mozambique in 2001 [15] and recently (2010) in Zambezia [18].

The ITN used are mostly long lasting types: the Olyset® (active ingredient: 2% permethrin) and PermaNets® (a.i: 50–55 mg/m2 deltamethrin) brands that were distributed in Zambezia since 2004 [16]. From 2004 to 2007, 559,126 LLIN were distributed in Mozambique, the bulk of these were in 2007 with majority of nets distributed in Milange district in Zambezia [16].

### Household survey and data preparation

**Figure 1:**
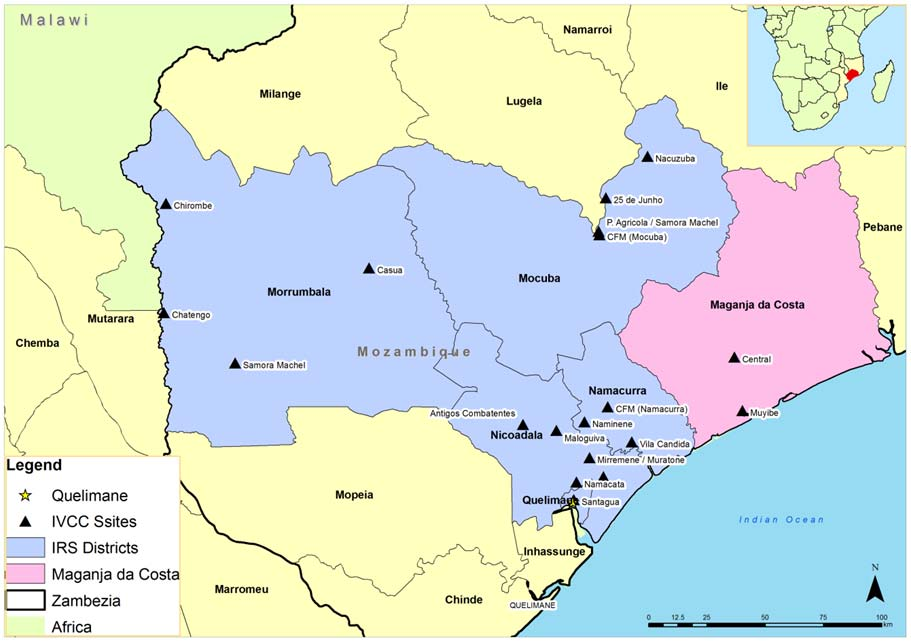
Map of Mozambique showing Zambezia province, the study site.

Malaria surveys were conducted annually in October in 2006, 2007 and 2008 in 19 sentinel sites (Figure 1) established for monitoring and surveillance of the malaria control program in 6 districts in Zambezia province. The surveillance was part of the Malaria Decision Support System project (MDSS) [19]. The survey instrument was adapted from the RBM - MERG Malaria Indicator Survey Household Questionnaire [20] to collect information on knowledge of preventive measures; IRS status of houses, net ownership and usage, household assets, house construction and ownership of farm animals. Children between ages of 1 to 15 years gave finger prick blood samples for a rapid diagnostic test (ICT; Global Diagnostics, South Africa), had their temperature taken, and history of fever recorded. Participants who tested positive were treated with Coartem® (Novartis) (Artemether and Lumefantrine) according to Mozambique's national malaria treatment guidelines.

History of fever was defined as parents reporting children who experienced fever in the past 4 weeks. Current fever was defined as body temperature ≥37.5°C taken during the survey. Head of household's education was categorised into those without formal education, those with primary/secondary education and those with tertiary education. Knowledge of malaria prevention was classified as adequate if the respondent was aware of malaria preventive measures in addition to IRS and ITN (use of repellents, coils or doom spray; burn leaves or cow dung; close windows and doors; use wire gauze; dispose cans and tyres; and drain water to reduce mosquito breeding). During the interview, respondent provided information on whether the net was treated or not but the ITN status was not verified by the interviewer. Given both IRS and ITN are malaria interventions applied in Zambezia, a composite variable constituting the 4 combinations of vector control interventions was used to develop the risk model as: 1) children in households without IRS and not sleeping under ITN, 2) children in houses with IRS and sleeping under ITN, 3) those protected by IRS but not sleeping under an ITN, and 4) those in unsprayed houses but sleeping under ITN. Socio economic status (SES) for each household was calculated from an index which combined ownership of assets (TV, radio, mobile phones, bicycles, motorcycles, cars and tractors) and house construction materials using principal component analysis [21], divided into quartiles, the 1^st^ quartile being the poorest. Houses were categorized into those covered by a grass roof and those covered with roof materials other than grass. The number of people in the household was grouped into 1–4 people, 5–7 people and 8–15 people.

### Statistical analysis

The following risk factors for malaria infection were investigated: whether the house had been sprayed (IRS) in the past 12 months, whether the child slept under a ITN the previous night, the child's sex and age, head of household's level of education, SES quartile, ownership of farm animal, house construction, knowledge of other malaria prevention practices, history of fever and current fever, and number of people in the house. Point estimates, confidence intervals, survey chi-square tests and estimates for odds ratio were derived taking account of the two-stage sample design by using the survey commands in Stata 11 [22] setting sentinel sites as the primary sampling unit (PSU). Data were merged to provide information for each child.

Crude odds ratio for the association between malaria infection and each explanatory variable were assessed one at a time using logistic regression. Variables with association (p<0.025) in the univariate analysis were considered for inclusion in a multivariable logistic regression model. The contribution of each covariate in the risk model was assessed by Wald tests and was included if p<0.05 after adjusting for confounders.

Sex and age were used as a priori potential confounders following practice adopted elsewhere [23].

### Ethics Approval

Ethics approval for the study was given by the Ministry of Health of Mozambique (Reg: 3622/IMS-2/DNS/06). Written informed consent was obtained from heads of households or responsible adults in each of the participating households.

## Results

### Characteristics of study population

A total of 2,748 households in 19 villages were surveyed in 2006, 2007 and 2008, with an average of 144 houses per sentinel site [range 99 to 175]. A total of 8,338 children from 1 to below 15 years of age participated, giving an average of 439 children/site.

The majority of children belonged to households whose head was not educated (42.4%) or had primary/secondary education (41.7%). The majority of children lived in houses covered with grass roof (77.6%) (Table 1). The proportion of children living in households with any farm animal was 47.5% (n = 3,957, 95%CI: 36.8–58.3). A high proportion of children were living in households with chickens (44.9%, n = 3,734, 95%CI: 34.7–55.5) compared to those living in houses with pigs (2.7%, n = 225, 95%CI: 1.4–5.1) or sheep (0.2%, n = 19, 95%CI: 0.05–1.1).

**Table 1:** Characteristics of children in Zambezia province.

	2006	2007	2008	Total
Characteristics	N(%)*	N(%)*	N(%)*	N(%)*
**Gender of children tested**				
Male	1233(47.8)	1366(48.2)	1297(45.2)	3896(47.0)
Female	1349(52.2)	1466(51.8)	1575(54.8)	4390(53.0)
**Age groups of children (years)**				
1–3	644(24.7)	690(24.3)	701(24.3)	2035(24.4)
4–6	777(29.7)	884(31.0)	988(34.2)	2649(31.8)
7–9	591(22.6)	651(22.9)	568(19.8)	1810(21.7)
10–15	598(23.0)	620(21.8)	626(21.7)	1844(22.1)
Total Children (below15 years)	2610	2845	2883	8338
**Children with history of fever past 4 weeks**				
No	1397(55.4)	1649(60.8)	1710(59.8)	4756(58.8)
Yes	1125(44.6)	1062(39.2)	1151(40.2)	3338(41.2)
**Children with current fever**				
No	2197(84.4)	2436(85.7)	2705(93.9)	7338(88.2)
Yes	405(15.6)	408(14.3)	173(6.1)	986(11.8)
**Children who slept under any net previous night**				
No	1778(70.2)	1808(66.6)	1867(65.7)	5453(67.4)
Yes	756(29.8)	907(33.4)	975(34.3)	2638(32.6)
**Children living in IRS house and sleeping under ITN**				
No IRS & not sleeping under ITN	682(31.5)	640(28.0)	559(23.6)	1881(27.6)
IRS & sleeping under ITN	269(12.4)	328(14.4)	450(19.0)	1047(15.4)
IRS & not sleeping under ITN	1027(47.5)	1168(51.1)	1192(50.4)	3387(49.7)
No IRS & sleeping under ITN	185(8.6)	149(6.5)	164(6.9)	498(7.3)
**Children living in household practicing other prevention measures than IRS & ITN**				
No other prevention practice	1007(38.6)	890(31.3)	850(29.5)	2747(33.0)
Any other prevention practices	1603(61.4)	1955(68.7)	2033(70.5)	5591(67.0)
**Size of household in which children live**				
1–4 people	510(20.3)	651(22.9)	656(22.7)	1817(22.0)
5–7 people	1367(54.3)	1616(56.9)	1587(55.1)	4570(55.5)
8–15 people	639(25.4)	572(20.2)	640(22.2)	1851(22.5)
**Children of parents with different level of education**				
None	721(32.3)	895(36.9)	1551(55.0)	3167(42.4)
Primary/Secondary	1141(51.1)	1073(44.2)	902(32.0)	3116(41.7)
Tertiary	371(16.6)	457(18.9)	367(13.0)	1195(15.9)
**Children living in household with different wealth index (SES)**				
1st quartile (poorest)	792(30.8)	640(22.5)	892(30.9)	2324(28.0)
2nd quartile	544(21.2)	660(23.2)	625(21.7)	1829(22.0)
3rd quartile	667(26.0)	782(27.5)	582(20.2)	2031(24.5)
4th quartile (wealthiest)	565(22.0)	763(26.8)	784(27.2)	2112(25.5)
**Roof material of house in which children live**				
Without grass roof	406(17.4)	518(20.1)	604(31.4)	1528(22.4)
With grass roof	1927(82.6)	2057(79.9)	1320(68.6)	5304(77.6)
**Children living in household with farm animals**				
Without farm animal	1425(54.6)	1490(52.4)	1466(50.8)	4381(52.5)
With farm animals	1185(45.4)	1355(47.6)	1417(49.2)	3957(47.5)

*N (%)  =  number of children and percentage in each category.

### Coverage of malaria control measures

The proportion of children who slept under any type of net the previous night increased from 29.8% (95%CI: 26.9%–32.8%) in 2006, to 34.3% (95%CI: 31.4%–37.4%) in 2008. Overall, 32.6% (95%CI: 30.9%–34.4%) of children were reported sleeping under any net the previous night (Table 1). Sixty five percent (95%CI: 63.6%–67.1%) of children were living in IRS treated houses ranging from 59.8% (95%CI: 56.4%–63.1%) in 2006 to 69.6% (95%CI: 66.3%–72.7%) in 2008.

As ITN and IRS campaigns were rolled out in Zambezia province, the proportion of children living in houses with IRS and sleeping under ITNs increased from 12.4% (95%CI: 7.6%–19.7%) in 2006 to 19.0% (95%CI: 13.4%–26.4%) in 2008 (Table 1). Overall, 15.4% (95%CI: 11.0%–21.0%) of children were living in IRS houses and sleeping under ITNs, 49.7% (95%CI: 39.6%–59.9%) were living in sprayed houses without sleeping under ITNs, 7.3% (95%CI: 4.2%–12.4%) were sleeping under ITNs in unsprayed houses and 27.6% (95%CI: 18.9%–38.3%) were neither sleeping under an ITN nor living in a sprayed house.

The proportion of children whose parents reported knowledge of other malaria preventive measures in addition to IRS or ITN was 67.0% (95%CI: 62.4%–71.4%), increasing from 61.4% (95%CI: 55.5%–67.1%) in 2006, to 70.5% (95%CI: 65.3%–75.2%) in 2008 (Table 1).

### Prevalence of malaria infection, ITN use and living in IRS houses

Overall prevalence of *P. falciparum* malaria infection in children (1–15 years) was 47.8% (95%CI: 38.7%–57.1%), of whom 15.8% (95%CI: 12.1%–20.3%) were co-infected with other plasmodial species. Malaria prevalence therefore refers to infections by *P. falciparum* alone or *P. falciparum* with other plasmodia. Malaria infection prevalence among children varied between the 19 villages ranging from 22.1% at Mocuba to 87.2% at 25-de-Junho. The proportion of infected children varied between years of survey from 51.9% in 2006, 60.3% in 2007, to 31.8% in 2008.

There was no evidence of difference in prevalence of malaria infection by sex (p = 0.27) but some evidence that it differed by household size (p = 0.065) and age group (p<0.012) ranging from 43.6% (95%CI: 41.2%–45.9%) in the 1–3 years age group to 51.7% (95%CI: 49.7%–54.1%) in the 7–9 years age group. More children presenting with current fever were infected with malaria (56.7%; 95%CI: 43.7%–68.9%) compared with those without fever (46.7%; 95%CI: 37.6–55.9, p = 0.037).

Malaria infections among children living in IRS houses and not sleeping under ITN were lower (OR = 0.6, 95%CI: 0.4–0.9; p = 0.013) than those in unsprayed (non IRS) houses and without sleeping under ITN. Children living in IRS houses and sleeping under ITN were additionally protected from malaria infection (OR = 0.4, 95%CI: 0.2–0.6, p = 0.001) relative to those not protected by either method. There was no evidence of a lower odds of malaria infection among children sleeping under ITN in unsprayed houses relative to those not protected by either methods (OR = 1.0, 95%CI: 0.7–1.5, p = 0.82) (Table 2).

**Table 2:** Association of malaria infection and other factors in Zambezia province.

		Children	Malaria infection			Univariate Analysis
Factors		N(%)*	%	P value	Trendˆ[Table-fn nt103]	OR§, 95%CI, p-value
**Gender of children tested**						
Male		3896(47.0)	49.0			1
Female		4390(53.0)	46.8	= 0.27		0.9(0.8–1.1)p = 0.395
**Age groups of children (years)**						
1–3		2035(24.4)	43.6			1
4–6		2649(31.8)	49.3			1.3(1.1–1.6)p = 0.001
7–9		1810(21.7)	51.7			1.5(1.2–1.8)p = 0.001
10–15		1844(22.1)	46.6	<0.012	0.013	1.2(0.9–1.5)p = 0.14
**Children with history of fever past 4 weeks**						
No		4756(58.8)	46.4			1
Yes		3338(41.2)	50.5	= 0.12		1.2(0.9–1.4) p = 0.05
**Children with current fever**						
No		7338(88.2)	46.7			1
Yes		986(11.8)	56.7	= 0.037		1.2(0.8–1.8) p = 0.69
**Children who slept under any net previous night**						
No		5453(67.4)	51.0			1
Yes		2638(32.6)	42.0	= 0.005		0.8(0.6–1.0)p = 0.048
**Children living in IRS house and sleeping under ITN**						
No IRS & not sleeping under ITN		1881(27.6)	60.6			1
IRS & sleeping under ITN		1047(15.4)	32.3			0.4(0.2–0.6)p = 0.001
IRS & not sleeping under ITN		3387(49.7)	46.2			0.6(0.4–0.9)p = 0.013
No IRS & sleeping under ITN		498(7.3)	59.4	= 0.0034		1.0(0.7–1.5)p = 0.82
**Children living in household practicing other prevention measures than IRS & ITN**						
No other prevention practice		2747(33.0)	54.4			1
Any other prevention practices		5591(67.0)	44.6	= 0.0023		0.8(0.6–0.9)p = 0.014
**Size of household in which children live**						
1–4 people		1817(22.0)	45.3			1
5–7 people		4570(55.5)	49.8			1.3(1.1–1.6)p = 0.001
8–15 people		1851(22.5)	45.7	= 0.065	0.845	1.3(1.0–1.6)p = 0.027
**Children if parents with different level of education**						
None		3167(42.4)	55.7			1
Primary/Secondary		3116(41.7)	44.6			0.5(0.4–0.6)p<0.0001
Tertiary		1195(15.9)	30.3	<0.0001	<0.0001	0.3(0.2–0.5)p<0.0001
**Children living in household with different wealth index (SES)**						
1st quartile (poorest)		2324(28.0)	54.4			1
2nd quartile		1829(22.0)	52.2			0.9(0.6–1.1)p = 0.286
3rd quartile		2031(24.5)	52.1			0.8(0.6–1.0)p = 0.058
4th quartile (wealthiest)		2112(25.5)	33.0	<0.0001	<0.0001	0.4(0.3–0.5) p<0.0001
**Roof material of house in which children live**						
Without grass roof		1528(22.4)	33.4			1
With grass roof		5304(77.6)	55.2	= 0.0004		1.8(1.2–2.7)p = 0.004
**Children living in households with farm animals**						
Chicken(s)	0	4591(55.2)	46.2			1
	≥1	3734(44.8)	49.8	= 0.35		1.2(0.9–1.5)p = 0.23
Goat(s)	0	7715(92.5)	47.4			1
	≥1	623(7.5)	53.1	= 0.25		1.3(0.8–1.9)p = 0.253
Sheep	0	8319(99.8)	47.9			1
	≥1	19(0.2)	31.6	= 0.12		0.5(0.3–1.0)p = 0.042
Cow(s)	0	8319(99.8)	47.8			1
	≥1	19(0.2)	55.6	= 0.75		1.0(0.1–7.6)p = 0.98
Pig(s)	0	8113(97.3)	47.2			1
	≥1	225(2.7)	70.1	= 0.0014		3.1(2.0–4.7)p<0.001
**Year of survey**						
2006		2610(31.3)	51.9			1
2007		2845(34.1)	60.3			1.5(1.1–2.0) p = 0.024
2008		2883(34.6)	31.8	<0.0001	<0.0001	0.4(0.3–0.7) p = 0.001

*N(%) = number of children and % in each category.

ˆ P-value based on a non-parametric test for trend.

§ Adjusted for age, asset index and year of survey.

### Risk factors for malaria infection

In univariate analysis, pig-keeping was associated with increased risk of malaria infection for children (OR = 3.1, 95%CI: 2.0–4.7, p<0.001), even after excluding 25 de Junho, the village with highest malaria infection prevalence (87.2%) and highest number of children living in households keeping pigs (29%, 65/225), there was still strong association between pig-keeping and malaria infection (OR = 2.6, 95%CI: 1.8–3.8, p<0.001). In the analysis including all villages, sheep keeping was associated with reduced risk of malaria infection (OR = 0.5, 95%CI: 0.3–1.0, p = 0.042) (Table 2), but the effect was less clear when Mocuba, a village with majority of children living in households keeping sheep (74%, 14/19) and one with the lowest malaria infection prevalence in children (22.1%) was removed from the analysis (OR = 1.3, 95%CI: 0.7–2.3, p = 0.418).

A majority of children (77.6%) were living in houses with a grass roof and these children were at elevated risk of malaria infection compared to those living in houses without a grass roof (OR = 1.8; 95%CI: 1.2–2.7, p = 0.004) (Table 2). Children living in large households with more than 8 people (OR = 1.3; 95%CI: 1.0–1.6, p = 0.027) were at higher risk of malaria infection compared to smaller household.

Children of educated parents (primary/secondary: OR = 0.5, 95%CI: 0.4–0.6, p<0.0001; tertiary: OR = 0.3, 95%CI: 0.2–0.5, p<0.0001) compared to uneducated parents, and those from high SES (wealthiest quartile) (OR = 0.4, 95%CI: 0.3–0.5, p<0.0001) compared to low SES (poorest quartile) were associated with lower odds of malaria infection (Table 2).

### The risk model for malaria infection

The multivariable logistic regression model was fitted sequentially starting with variables having an association with malaria infection (p<0.025) (Table 2). Age of child, household SES, year of survey, and the intervention variable (ITN, IRS status of house or both) were included a priori in the model. The model demonstrated that pig-keeping, living in houses with a grass roof, large household size, low parents education level and current fever were associated with an increased risk of malaria infection (Table 3).

**Table 3:** Multivariable logistical regression model of risk factors for malaria infection in Zambezia province.

Factors		OR*(95%CI)	Wald P-value	Adjusted P-value*
**Age groups of children (years)**				
1–3		1		
4–6		1.3(1.1–1.5)	= 0.01	
7–9		1.4(1.1–1.7)	= 0.016	
10–15		1.3(1.0–1.6)	= 0.056	
**Children with current fever**				
No		1		
Yes		1.2(1.0–1.5)	= 0.076	
**Living in IRS houses and sleeping under ITN**				
No IRS & not sleeping under ITN		1		
IRS & sleeping under ITN		0.5(0.3–0.7)	= 0.001	
IRS & not sleeping under ITN		0.6(0.4–0.9)	= 0.018	
No IRS & sleeping under ITN		1.2(0.9–1.8)	= 0.238	0.009
**Size of household in which children live**				
1–4 people	1			
5–7 people	1.6(1.3–2.0)		<0.0001	
8–15 people	1.6(1.3–2.1)		= 0.001	0.0022
**Children of parents with different level of education**				
None	1			
Primary/secondary	0.6(0.5–0.7)		<0.0001	
Tertiary	0.4(0.3–0.6)		<0.0001	<0.0001
**Children living in household with different wealth index (SES)**				
1st quartile (poorest)	1			
2^nd^ quartile	0.9(0.7–1.2)		= 0.50	
3^rd^ quartile	0.9(0.7–1.3)		= 0.723	
4th quartile (wealthiest)	0.5(0.4–0.7)		<0.0001	
**Roof material of house in which children live**				
Without grass roof	1			
With grass roof	1.7(1.3–2.4)		= 0.002	
**Children living in household with pig**				
No pig	1			
Own pig(s)	3.2(2.1–4.9)		<0.0001	
**Year of survey**				
2006	1			
2007	1.4(1.0–2.1)		= 0.047	
2008	0.4(0.3–0.7)		= 0.003	

OR* adjusted for age, year of survey and wealth index. Estimates of OR* for covariates not related with farm animals were done with pig variable in the model.

*P-value derived from Wald test adjusted for the combine effect of categories in the variable.

In the final model, the risk of malaria infection was much lower among children living in an IRS household and sleeping under an ITN (OR = 0.5, 95%CI: 0.3–0.7) and those living in IRS household and not sleeping under ITN (OR = 0.6, 95%CI: 0.4–0.9) relative to those living in households without either of the two interventions. There was no evidence that sleeping under ITN in an unsprayed house was protective against malaria infection (OR = 1.2, 95%CI: 0.9–1.8, p = 0.238). Children of educated parents were at lower risk of malaria infection (primary/secondary education: OR = 0.6, 95%CI: 0.5–0.7, and tertiary education: OR = 0.4, 95%CI: 0.3–0.6) relative to those with no education.

On the other hand, pig-keeping (OR = 3.2, 95%CI: 2.1–4.9, p<0.0001) was associated with increased risk of malaria infection in children compared to those living in households without pigs. Malaria infection was also associated with children living in houses with a grass roof (OR = 1.7, 95%CI: 1.3–2.4, p = 0.002) relative to children living in houses with a non-grass roof. Overall the risk of malaria infection increased for children living in large households (5–7 people: OR = 1.6, 95%CI: 1.3–2.0, p<0.0001; ≥8 people: OR = 1.6, 95%CI: 1.3–2.1, p = 0.001) relative to those living in small household (1 to 4 people). Children with current fever were more likely to be infected (OR = 1.2, 95%CI: 1.0–1.5, p = 0.076) with malaria than those without fever.

There was no evidence that knowledge of prevention measures other than IRS and ITN was independently associated with risk of infection. The odds of malaria infection for sheep keeping (OR = 1.4, 95%CI: 0.9–2.1, p = 0.158) was in the same direction as pig keeping (OR = 3.2, 95%CI: 2.1–4.9, p<0.001) when sheep keeping was included in the model.

## Discussion

In Zambezia province, malaria prevalence is high (47.8%) in children of all age groups below 15 years confirming that malaria remains a major cause of illness during childhood. Likewise, a survey of malaria across Mozambique reported overall malaria prevalence of 49% and relatively high age-specific malaria infection ranging from 39% for 7–10 years to 55% for children 1–2 years [24].

Children living in IRS treated houses and sleeping under an ITN (OR = 0.5, 95%CI: 0.3–0.7, relative to those not protected by either method) were at lower risk of malaria infection than children living in IRS houses and not sleeping under ITN (OR = 0.6, 95%CI: 0.4–0.9, relative to those not protected by either method), p = 0.028. The combined protective effect of IRS and ITN has been reported previously [9], [25]. Although the impact of ITN on reducing malaria morbidity and mortality has been confirmed in robust randomised control trials [26], these surveys did not show evidence of protection against malaria infection in children sleeping under ITN in unsprayed houses (OR = 1.2, 95%CI: 0.9–1.8) compared to those not sleeping under ITN in unsprayed houses. This observation could be due to chance, related to irregular compliance or improper use of nets [27], or the result of reverse causation with nets being targeted at communities that have the highest risk of malaria and households burdened by repeated episodes of malaria being more likely to use nets. Recent evidence of pyrethroid insecticide resistance, detected in *An. funestus* [18] in Zambezia could have reduced the protective effect of ITNs, particularly if they were in poor condition [28].

Knowledge of malaria preventive measures other than IRS and ITN (use of repellents, coils or doom spray, burn leaves or cow dung, etc), did not appear to have any protective effect on children, emphasising that conventional methods such as IRS and use of ITNs should be encouraged for public heath use of malaria control.

Keeping farm animals, in particular pigs and to lesser extent sheep was associated with increased risk of malaria infection even after adjusting for confounders such as household wealth, suggesting a possibility that certain farm animals may attract malaria vectors to the houses. Several studies have evaluated the relationship between malaria infection and keeping animals with conflicting results [29], [30]. *Anopheles arabiensis*, attracted to animals and displaying opportunistic feeding behaviour [6], was found in Zambezia in low density at the time of the surveys compared to *An. gambiae s.s* and *An. funestus*. Although the latter are inherently endophilic, feeding mainly on human hosts [31], [32], there are reports of *An. funestus* being not necessarily entirely anthropophagic [33], which would explain their attraction to houses with animals. . Further investigation into the relationship between farm animals and malaria infections occurring at different seasons may elucidate possible trends.

Malaria risk can vary widely between villages or even households [34] due to their specific characteristics that may facilitate human–mosquito contacts. The majority of children (78%) in Zambezia are living in houses with a grass roof which was associated with a high odds of malaria infection compared to those living in houses with other types of roof. This association confirms previous findings, from Eritrea, of increased risk of malaria infection in houses made with similar material providing microenvironments conducive for mosquitoes, extending their chance of human contact opportunities and survival [35], [36]. House modifications and improvements have been associated with a reduction of anopheline mosquitoes entering the house [37] and a decrease of malaria infection prevalence [38] and anaemia [39]. Whilst such interventions are likely to be costly, they should be considered as part of long term preventive measures. In the meantime it is important that IRS spray teams are trained to spray not only the interior walls of houses, but the ceilings and underside of roofs as well, particularly where grass is used for roofing.

Education and smaller household size were associated with lower odds of malaria infection. Educated parents are more likely to encourage their children to sleep under a net because they are better informed and living in smaller household can provide adequate space to hang nets.

The likelihood of a child presenting with fever to be infected with malaria infection was high, with 56.7% of fever cases having malaria infection compared to 46.7% in none fever cases. Other studies have confirmed that malaria infections remain a major cause of febrile illness during childhood [24], [40]. Nevertheless, 43% of fever cases were not infected with malaria parasites, highlighting the need to diagnose and treat all fevers among children.

In this study several limitations should be noted. Firstly, the surveys were conducted in October coinciding with the onset of the rainy season with still low mosquito densities and malaria transmission. Also the study does not provide all information on factors determining the risk of malaria infection throughout the year. Secondly, survey sites are mainly located in the southern part of Zambezia province therefore the results are not necessarily representative of the whole province or the country. Thirdly, the malaria rapid tests are subject to limitations in sensitivity and specificity which could have lead to an underestimation or overestimation of infection prevalence since no blood slides were taken for validation purposes [41], [42]. Some answers to question such as enquiring about sleeping under ITNs were reported by the parents and not observed by interviewers. Likewise, it was not possible for the interviewers to verify the ITN status of nets during the survey. Finally, malaria infection is likely to be over-estimated in groups of children remaining at home (possibly sick from malaria) and underestimated in children not found at home (likely to be healthy children, hence attending school).

In conclusion, the malaria burden among children 1 to under 15 years of age is high in Zambezia province. Consequently, health education and treatment should not only target vulnerable groups (children under 5 and pregnant women), but all the age groups. Since almost half (43%) of fever cases were not infected with malarial parasites, this has implications on policy of fever treatment, particularly in rural areas where all febrile cases may be wrongly treated as clinical malaria. Children living in IRS sprayed houses, or living in a sprayed house and sleeping under an ITN were at much lower odds of malaria infection than children living in unsprayed houses and not sleeping under ITN. The risk of malaria infection was associated with keeping farm animals, particularly pigs and living in houses with grass roofs. In addition to ensuring high coverage of IRS, which should include the spraying of grass roofs, and promotion of the use of ITNs, malaria control programs should consider advising owners of farm animals to ensure a reasonable separation between animal sheds and sleeping areas for humans. Households with increased risk of infection, such as those keeping animals, and those with grass roofs should be particularly targeted for ITN use.
